# Effects of hemodynamic monitoring using a single-use transesophageal echocardiography probe in critically ill patients – study protocol for a randomized controlled trial

**DOI:** 10.1186/s13063-018-2714-4

**Published:** 2018-07-06

**Authors:** Luca Cioccari, Bjoern Zante, Andreas Bloch, David Berger, Andreas Limacher, Stephan M. Jakob, Jukka Takala, Tobias M. Merz

**Affiliations:** 1 Department of Intensive Care Medicine, University Hospital, and University of Bern, 3010 Bern, Switzerland; 2 CTU Bern, and Institute of Social and Preventive Medicine (ISPM), University of Bern, Bern, Switzerland

**Keywords:** Circulatory shock, Intensive care unit, Hemodynamic monitoring, Echocardiography

## Abstract

**Background:**

Hemodynamic instability is one of the leading causes of intensive care unit (ICU) admission. Early stabilization of hemodynamics is associated with improved outcome. The monitoring used to guide hemodynamic support may influence the time needed to achieve stable hemodynamics. Visualization of the heart using echocardiography offers the advantage of direct measurement of cardiac volumes and ventricular function. A miniaturized monoplane transesophageal echocardiography (TEE) probe was developed, allowing for almost continuous qualitative hemodynamic TEE assessment (hTEE) after brief bedside training. The primary objective of the study is to assess whether hemodynamic monitoring using the hTEE technology shortens time to resolution of shock in ICU patients in comparison to standard monitoring using a central venous catheter, pulmonary artery catheter, or conventional echocardiography.

**Methods:**

Five hundred consecutive subjects with circulatory shock (low mean arterial blood pressure (MAP) and signs of organ hypoperfusion) at the time of ICU admission are included in the study. The subjects are randomly assigned to one of four groups using a 2 × 2 factorial design stratified by method of hemodynamic monitoring (hTEE vs standard hemodynamic monitoring) and frequency of hemodynamic assessments (minimum every 4 h vs standard of care). The primary study outcome is the time from study inclusion to resolution of circulatory shock, defined as MAP >  60 mmHg for ≥ 4 h after discontinuation of vasopressors and inotropes. The hTEE monitoring consists of the acquisition of three defined echocardiography views: Transgastric mid-esophageal short axis with measurement of fractional area change of left ventricle, mid-esophageal four-chamber view with measurement of the ratio of right to left ventricular area, and mid-esophageal ascending aortic short-axis view with measurement of the superior vena cava collapsibility index. In the control groups, monitoring modalities, including conventional TTE and TEE but not hTEE, are at the discretion of the treating physician. The interpretation of hemodynamic monitoring and the subsequent changes in patient management are recorded after each hemodynamic assessment. Differences in the primary and further secondary time-to-event outcomes will be assessed using a competing risk model accounting for the competing risk of death.

**Discussion:**

The effect of using echocardiography as a monitoring modality on relevant patient outcomes has not been established so far. The study at hand may be one of the first trials to provide detailed data on effectiveness and safety of echocardiography to guide treatment in patients with circulatory shock.

**Trial registration:**

ClinicalTrials.gov, ID: NCT02048566. Registered on January 29, 2014.

**Electronic supplementary material:**

The online version of this article (10.1186/s13063-018-2714-4) contains supplementary material, which is available to authorized users.

## Background

Hemodynamic management of critically ill patients is a constant challenge in the intensive care unit (ICU). Available monitoring parameters to guide hemodynamic management consist of measurements of pressures (systemic and pulmonary artery pressures, central venous pressure) and flow (cardiac output measurements). However, cardiac filling pressure data have known limitations and might not accurately represent cardiac preload and contractility [1]. To date, continuous or sequential recording of cardiac output parameters is limited to pulse contour analysis and indicator-dilution techniques [2]. These methods allow only an incomplete characterization of the patient’s cardio-circulatory status and comparisons of different measurements of cardiac function parameters are reported to trend differently in response to therapy and to show limited inter-device agreement [3]. Hemodynamic management of critically ill patients based on these parameters might, therefore, not be optimal and delay the stabilization of the patient, leading to worse outcomes and increased use of resources [4].

Echocardiography is an established tool to evaluate the causes of hemodynamic instability in ICU patients by the visualization of cardiac chambers, valves and pericardium and of cardiac functional abnormalities [5, 6]. Transthoracic (TTE) or transesophageal (TEE) echocardiography can be used as a first-line approach for a quick and focused examination [7]. However, the training necessary to reliably perform focused echocardiography is substantial [8, 9] and the method is not readily available for every intensivist. A repeated echocardiographic assessment could potentially provide useful additional information resulting in more rapid resolution of hemodynamic instability [10]. Monitoring hemodynamics with conventional TTE and TEE is not feasible due to a lack of time and availability of appropriately trained staff.

A miniaturized monoplane TEE probe that can be left inserted in the esophagus for up to 3 days has been recently developed. Qualitative hemodynamic TEE assessment (hTEE) using the miniaturized probe allows for almost continuous echocardiographic monitoring of unstable ICU patients [11], and can provide useful additional information for hemodynamic management when compared to standard, non-ultrasound-based monitoring modalities [10]. The feasibility of such qualitative hTEE by intensivists after a 6-h bedside training has been demonstrated [12]. However, as of yet, studies assessing the impact of hemodynamic monitoring by hTEE on relevant patient outcomes are not available. Given the associated costs for the hTEE device and the ultrasound probes and the additional resource requirements for training and use, the efficacy and efficiency of hTEE monitoring in comparison to standard monitoring should be established.

## Methods

### Hypothesis

The primary study hypothesis is that hemodynamic monitoring using hTEE in critically ill patients receiving cardiovascular support allows for a shorter time to resolution of circulatory shock compared to standard ICU monitoring alone, using a central venous catheter, pulmonary artery catheter, or conventional TTE and TEE. In this context, we define time to resolution of shock as the time from randomization to the time point of resolution of circulatory shock (first time point fulfilling the definition mean arterial pressure (MAP) ≥ 60 mmHg for at least 4 h after discontinuation of vasopressors and inotropes). The secondary hypothesis is that hemodynamic assessment at least every 4 h results in shorter time to resolution of circulatory shock as compared to standard hemodynamic assessment (at least once per nursing shift).

### Objectives

The primary objective of the study is to assess whether hemodynamic monitoring using the hTEE technology shortens time to resolution of shock in ICU patients in comparison to standard monitoring using a central venous catheter, pulmonary artery catheter, or conventional echocardiography. Secondary objectives include whether hemodynamic assessment at mandated 4-hourly intervals shortens the time to resolution of shock in comparison to standard assessment intervals, as well as the safety and tolerability of the hTEE probe.

### Outcomes

The primary study outcome is the time from study inclusion to resolution of circulatory shock (as defined above). The secondary outcomes include the time to resolution of clinical signs of shock (capillary refilling time < 3 s, urine output > 0.5 mL/kg/h for at least 4 h, blood lactate < 2 mmol/L)), the length of time on organ support (mechanical ventilation, renal-replacement therapy), length of stay (LOS) in the ICU and in hospital, ICU and hospital mortality, and the use of conventional hemodynamic monitoring (pulmonary artery catheter, central venous catheter, conventional echocardiography) and occurrence of SAE. A detailed description of study outcomes and adverse events is included in Additional file 1.

### Trial design

This is a randomized, open label, 2 × 2 factorial design, controlled clinical trial comparing the effect of hemodynamic monitoring using the hTEE technology (ImaCor ClariTEE system IMACOR, New York, NY, USA) or standard hemodynamic monitoring in a single center. Patients with circulatory shock, defined as persistent hypotension despite adequate fluid resuscitation and signs of hypoperfusion or organ dysfunction (at least one of the following: capillary refilling time 3 s or longer, urine output < 0.5 mL/kg for at least 1 h, lactate > 2 mmol/L) are included into the study at the time of ICU emergency admission. Subjects are randomized into four groups using a 2 × 2 factorial study design. The four groups are stratified by method of hemodynamic monitoring (hTEE vs standard hemodynamic monitoring) and frequency of hemodynamic assessments (protocolized maximal interval of 4 h vs standard monitoring intervals with maximal interval of 8 h). Assessed factors are use of hTEE monitoring vs conventional monitoring and use of protocolized intervals for hemodynamic assessment vs standard assessment intervals. The Standard Protocol Items Recommendations for Interventional Trials (SPIRIT) checklist is attached as Additional file 2.

### Study setting

The study is performed at the Department of Intensive Care Medicine of the University Hospital Bern, a 60-bed ICU in a 960-bed tertiary care center in Switzerland.

### hTEE operators

ICU specialists perform the study examinations. All hTEE operators receive a total of 4 h training in the use of hTEE by an experienced operator. Training includes an introduction to the method and a demonstration of the device use in the context of a presentation, followed by one-to-one bedside training of all necessary skills to use the hTEE device for acquiring and interpreting images in the study context.

### Patient eligibility criteria

All ICU patients are screened at the time of ICU admission for eligibility for the study. All ICU admissions of the last 24 h are additionally and independently screened to identify missed study patients each day at 8 a.m. to achieve adequate patient enrollment. Subjects of 18 years of age or older who require mechanical ventilation through an endotracheal, naso-tracheal, or tracheostomy tube with circulatory shock of any cause are included in the study. Circulatory shock is defined as follows:

- Systemic mean arterial blood pressure < 60 mmHg (or < 80 mmHg if the patient has baseline hypertension) for more than 30 min despite adequate fluid resuscitation (minimum of 20 mL/kg of crystalloids) or maintaining the systemic mean arterial blood pressure ≥ 60 mmHg requires any dose of vasopressors (norepinephrine, vasopressin) or inotropes (epinephrine, dobutamine, milrinone, aminophylline)
- Signs of hypoperfusion or organ dysfunction (at least one of the following: capillary refilling time 3 s or longer, urine output < 0.5 mL/kg for at least 1 h, lactate > 2 mmol/L)

### Patient exclusion criteria

Exclusion criteria consist of pathologies of the upper gastrointestinal tract (unrepaired trachea-esophageal fistula, history of prior esophageal or gastric surgery precluding the use of TEE, esophageal obstruction, stricture, varices or diverticulum, esophageal or gastric perforation or esophageal bleeding, vascular ring, aortic arch anomaly with or without airway compromise, recent oropharyngeal surgery) and severe coagulopathy (thrombocyte count less than 30 × 10^9^/L or INR > 3). Additionally, patients with cervical spine injury or anomaly, patients after elective ICU admission after planned surgery and patients with cardiac assist devices (intra-aortal balloon pump, ventricular assist devices, extra-corporeal membrane oxygenation) are not included in the study.

### Allocation

The allocation sequence was generated before commencement of the study by ICU research staff using computer-generated random numbers with randomly varying block sizes of 4, 8 and 12 [13]. Allocations concealment is ensured using sequentially numbered, identical, opaque, sealed envelopes. Patients are randomized and enrolled by the admitting on-call ICU specialist after reviewing the inclusion and exclusion criteria and after obtaining confirmation by a physician who is not participating in study that the interests of the patient are safeguarded. Due to logistical reasons (two hTEE devices available for the study) inclusion of a maximum of two patients into the groups applying hTEE is possible at the same time. Randomization and recruitment is interrupted as soon as a second patient is randomized into a treatment group in which hTEE is applied. Randomization and recruitment is restarted as soon as at least one hTEE device is available for further patients. Blinding of health care providers is not feasible which means that all clinical staff caring for the patients are aware of the allocation during the study period.

### Interventions and study procedures

The subjects are assigned to one of four groups stratified by method of hemodynamic monitoring (hTEE vs control hemodynamic monitoring) and frequency of hemodynamic assessments (protocolized maximal interval of 4 h vs standard monitoring intervals with maximal interval of 8 h) (Table 1):

**Table 1:** Study groups

Study group	Hemodynamic monitoring	Monitoring interval	*n*
hTEE protocolized monitoring (hTEEPM)	• hTEE-guided hemodynamic management • Additional hemodynamic monitoring of choice of the treating physician	• Study inclusion • Time of occurrence of new organ system deterioration • At least every 4 h	125
hTEE standard monitoring (hTEESM)	• hTEE-guided hemodynamic management • Additional hemodynamic monitoring of choice of the treating physician	• Study inclusion • Follow-up measurement time points at the discretion of the treating physician • At least every 8 h (standard of care in our ICU)	125
Control protocolized monitoring (ControlPM)	• Any hemodynamic monitoring of choice of the treating physician except hTEE	• Study inclusion • Time of occurrence of new organ system deterioration • At least every 4 h	125
Control standard monitoring (ControlSM)	• Any hemodynamic monitoring of choice of the treating physician except hTEE	• Study inclusion • Follow-up measurement time points at the discretion of the treating physician • At least every 8 h (standard of care in our intensive care unit (ICU))	125

In patients randomized to echocardiography-guided hemodynamic management (hTEEPM and hTEESM) the ImaCor ClariTEE system (IMACOR, New York, NY, USA) is installed at the time of study inclusion by the ICU specialist in charge of the patient. The device produces a single-plane two-dimensional image and has color Doppler capability. The hTEE probe is a 5.5-mm detachable probe; due to its small size, it can remain in situ for up to 72 h and, therefore, allows for reassessment of the patient’s hemodynamic progress and the effect of selected interventions at any time. Before insertion, the probe is connected to a dedicated echocardiographic system, which allows the recording of digital loops and the performance of basic two-dimensional measurements of areas and distances. Additional hemodynamic monitoring modalities are restricted to techniques routinely used at the study center (central venous catheter, pulmonary artery catheter, and conventional echocardiography) and can be used at the discretion of the ICU specialist in charge. After positioning of the hTEE probe, the following three hTEE standard views and measurements are acquired and performed:

- transgastric mid-esophageal short-axis view (TG mid SAX); fractional area change (FAC) of left ventricle (LV)
- Mid-esophageal four-chamber view (ME four-chamber); ratio of right ventricular (RV) to left ventricular area (RVEDA/LVEDA ratio)
- Mid-esophageal ascending aortic short-axis view (ME asc aortic SAX); collapsibility index of superior vena cava (SVC CI)

Left ventricular areas at end-systole (LVESA) and at end-diastole (LVEDA) are measured from the TG mid SAX view, the fractional area change (FAC) of the left ventricle (LV) is calculated as LVEDA/LVESA and used to grade left ventricular ejection fraction as normal (FAC > 50%), moderately decreased (FAC 40–50%) or severely decreased (FAC < 40%). Similarly, calculation of the RV/LV ratio is performed by measuring left and right ventricular areas at end-diastole in the ME four-chamber view. A ratio > 0.6 is used as indicator of right ventricular dysfunction. The collapsibility of the superior vena cava (SVC) is rated by calculating the collapsibility index, i.e., the inspiratory decrease in SVC diameter. The index is determined as (maximal diameter on expiration – minimal diameter on inspiration)/maximal diameter on expiration, expressed as a percentage [14]. A threshold of > 35% is used to distinguish between the presence and absence of hypovolemia. After image acquisition, the ICU specialist answers the following questions about quantification/interpretation of all hemodynamic monitoring and subsequent changes in hemodynamic management using a study case report form (CRF) (Table 2):

**Table 2:** Quantification/interpretation of hemodynamic monitoring and changes in hemodynamic management

Quantification/interpretation of all hemodynamic monitoring	Changes in hemodynamic management based on information acquired by hemodynamic monitoring?
• Systolic LV function: normal – moderately decreased – severely decreased? • RV dysfunction: present –absent? • Hypovolemia: present – absent? • Clinically significant pericardial effusion: present – absent? • Was the information acquired by hemodynamic monitoring useful to guide hemodynamic management? Yes – no? • Is there need for further monitoring? Yes – no?	• No changes • Additional fluids given • Start/increase of inotropes (epinephrine, dobutamine, milrinone, aminophylline) • Stop/decrease inotropes (epinephrine, dobutamine, milrinone, aminophylline) • Start/increase of vasopressors (norepinephrine, vasopressin) • Stop/decrease of vasopressors (norepinephrine, vasopressin) • Drainage of pericardial effusion • Other (to be specified)

*RV* right ventricular, *LV* left ventricular

In patients randomized to conventional hemodynamic management (ControlPM and ControlSM) hemodynamic monitoring is established at the discretion of the ICU specialist in charge. The use of hTEE is excluded in this group, whereas the use of conventional TTE or TEE is permitted. After hemodynamic assessment, the ICU specialist answers the same questions about quantification/interpretation of all hemodynamic monitoring and subsequent changes in hemodynamic management as in groups hTEEPM and hTEESM (Table 2).

Study procedures and measurements in all groups are performed until the primary outcome (resolution of shock) occurs or for a maximum of 72 h. Criteria for discontinuing hTEE monitoring are any occurrence of complications potentially attributed to the use of hTEE such as oral, pharyngeal, esophageal, or gastrointestinal bleeding or injury and cardiac arrhythmias (supraventricular/ventricular tachycardia, atrioventricular block, asystole) attributed to the presence of an hTEE probe by the treating physician. For the control groups (ControlPM, ControlSM) criteria for discontinuing the allocated intervention have not been defined as they receive standard intensive care. Adherence to the study protocol is monitored for each included patient by daily monitoring of completion of study CRF by the research staff. Concomitant care for all study groups consists of standard interventions in the context of the routine care of ICU patients as per the decision of the treating ICU specialist. The ICU uses a cardiovascular management protocol to guide hemodynamic stabilization [15]. Commonly used monitoring modalities consist of measurement of arterial and central venous pressure, use of pulmonary artery catheters with continuous monitoring of cardiac output and mixed venous saturation, and conventional echocardiography. The standard vasopressor is norepinephrine; standard inotropes are dobutamine, milrinone and epinephrine. Pulse-contour analysis modalities are not applied.

### Participant timeline

**Fig. 1:**
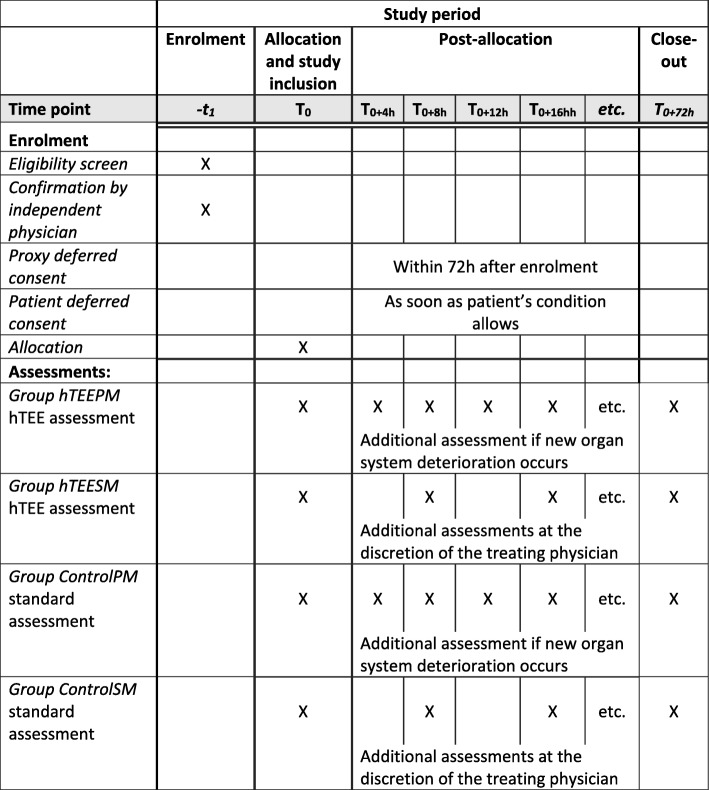
Participant timeline

The time schedule of enrollment, interventions and assessments is outlined in Fig. 1. Recruitment of study subjects started in March 2014 and is ongoing.

### Sample size

Sample size calculation is based on a retrospective analysis of a sample of 159 patients admitted to our ICU during a 3-month period, which fulfilled the study entry criteria. Median time to resolution of circulatory shock, defined as mean systemic blood pressure > 60 mmHg and resolution of clinical signs of shock (capillary refilling time < 3 s, urine output > 0.5 mL/kg/h for at least 4 h, blood lactate < 2 mmol/L), in this sample was 18.5 h (Fig. 1). We used the Stata command artsurv to calculate the sample size [16]. We applied the unweighted log-rank test and derived expected probabilities of hemodynamic stabilization and loss-to-follow up (i.e., death) over time from the retrospective analysis. To identify a clinically relevant reduction of time to resolution of circulatory shock of 25% (i.e., from 18.5 to 14.0 h) we calculated a required sample size of 458 patients to achieve a power of 80% at a two-sided alpha level of 0.05 for the main effect (comparison of monitoring with/without hTEE). In order to allow for study drop out, the inability to consent, or the withdrawal of consent, we choose a sample size of 125 patients for each of the four groups or 500 patients in total. Patient recruitment will be continued until a total of 500 patients with a complete study follow-up, and in which consent for study inclusion has been obtained by the patient, are included into the study.

### Data collection methods

In all study patients, hemodynamic parameters, use of vasopressors/inotropes as continuous intravenous infusions, parameters determined by organ support (mechanical ventilation, renal-replacement therapy) and results of blood sampling are registered automatically by the electronic patient data management system (PDMS) as part of the routine patient data collection. Capillary refill time (hourly) and ICU resource use (accumulated TISS points, hourly) and urinary output (2-hourly) are part of the routine data collection performed by the bedside ICU nurse; these data are manually entered into the PDMS. LOS in hospital and hospital mortality are extracted from the hospital database. Blood lactate levels are measured in study patients at least every 2 h. A paper-based study CRF is the primary data collection instrument for the study. All study data necessary for statistical analysis according to the SAP are recorded in the CRF. All data requested on the CRF are recorded during hemodynamic assessment. If a space on the CRF is left blank because the procedure was not done or the question was not asked, “N/D” is written instead. If an item is not applicable to the individual case, “N/A” is written. All corrections must be initialed and dated. All hTEE echocardiography loops are recorded and saved for independent and blinded off-line review.

### Data management

Data are entered into a web-based electronic CRF. Study subjects are assigned an individual identifying study code on all data documents, which does not contain identifying information. The investigators keep a separate documentation that links the study code to subjects’ identifying information locked in a separate location and restrict access to this document to research staff. CRF and electronic data are archived for 10 years.

### Statistical methods

Continuous baseline and procedural variables will be displayed as mean (standard deviation) for normally and median (quartiles) for non-normally distributed data, categorical variables as number (percentage). The primary analysis will be an intention-to-treat (ITT) analysis, i.e., all patients will be analyzed as randomized. We will first test the primary hypothesis, the difference in time to hemodynamic stabilization between the hTEE and standard group. Second, we will evaluate the difference between the protocoled and standard monitoring. Differences in the primary outcome will be assessed using the Fine-Gray competing risk model accounting for the competing risk of death [17]. The model that compares the method of monitoring (hTEE vs standard) will be adjusted for the frequency of monitoring (protocolizedvs standard), the model that compares the frequency of monitoring for the method of monitoring. We will also test for an interaction between the method (hTEE vs standard) and frequency of hemodynamic monitoring (protocolized vs standard) in order to assess if effects are different depending on the method and frequency of monitoring. We will enter an interaction term in the model described above. If there is significant interaction, we will present effects separately in subgroups.

The secondary outcome, time to death, will be evaluated using Cox proportional-hazards regression. Other secondary time-to-event outcomes will be analyzed using competing risk models as described above. Binary outcomes will be analyzed using logistic regression, continuous score data using linear regression. We will also perform a secondary per-protocol analysis excluding patients that did not receive the allocated interventions or had major protocol violations. Statistical analyses will be described in more detail in a statistical analysis plan (SAP).

### Data monitoring

The trial is externally monitored (Clinical Trials Unit, Bern, Switzerland) in accordance with Good Clinical Practice (GCP) standards.

### Monitoring harm

Occurrence of adverse events due to prolonged hemodynamic impairment and treatment with vasopressors/inotropes is registered, including ventricular tachycardia, ventricular fibrillation, atrial fibrillation, myocardial infarction as defined in [18], skin necrosis, stroke, secondary bowel or limb ischemia, secondary infections (catheter-related infections, ventilator-associated pneumonia VAP, new onset of sepsis) and gastrointestinal bleeding complications. Additionally, any occurrence of complications potentially attributed to the use of hTEE such as oral, pharyngeal, esophageal, or gastrointestinal bleeding or injury, cardiac arrhythmias occurring while hTEE probe is in place (supraventricular/ventricular tachycardia, atrioventricular block and asystole) and need for additional sedation and/or muscle relaxants for hTEE probe placement and hTEE assessment is recorded. The study includes critically ill patients with circulatory shock. Depending on the reasons for hemodynamic impairment and comorbidities a mortality rate of up to 50% has to be expected [19]. In accordance with current guidelines, serious adverse events (SAE) and serious adverse device effect (anticipated or unanticipated) will be thoroughly investigated to determine causality and reported to the appropriate authorities (Kantonale Ethikkommission Bern) if a relation between SAE and study procedures is considered unlikely, likely, or certain, or in the event of death of the patient. No SAE report is made if a connection between SAE and study procedures can be excluded. Subjects experiencing any trial-related adverse events will receive the best possible care, including follow-up visits as clinically indicated.

### Confidentiality

Information about study subjects is kept confidential. A signed subject authorization is part of the informed consent documents, informing the subject what protected health information (PHI) is collected from subjects in this study, who will have access to the information and the rights of a research subject to revoke their authorization for use of their PHI. All data are entered into a dedicated study data management system. Study subjects are assigned an individual identifying study code on all data documents (e.g., completed questionnaire) which does not contain identifying information. The investigators keep a separate documentation that links the study code to subjects’ identifying information in a separate location.

## Discussion

The targeted patient population fulfills the definition of circulatory shock, representing the most severely ill patients with plausibly the highest possible chance to show a difference between the intervention arms. Echocardiography has proven a useful tool to identify reversible causes of shock and to monitor left and right ventricular function [20]. However, despite several class 1 recommendations for its use in the ICU, the effect of echocardiography as a monitoring modality on relevant patient outcomes has not been studied in large randomized trials so far. The study at hand aims at establishing the effect of frequent hemodynamic TEE monitoring using a miniaturized probe that allows minimizing invasiveness of TEE examinations. The study may be one of the first trials to bridge the gap between evidence and clinical practice and to provide detailed data on efficacy and safety of echocardiography to guide treatment in patients with circulatory shock.

### Strengths and limitations

To our knowledge, this is the largest randomized controlled trial of the effect of hTEE-monitoring in critically ill patients with circulatory shock. The primary outcome is objective and verifiable, and the statistical analysis adjusts for frequency and method of monitoring, including testing for interaction, in order to assess if any difference in outcome depends on the method or on the frequency of monitoring. The feasibility of hTEE monitoring has been previously established and the technology has shown to provide additional valid information for the management of critically ill patients. Our study has some limitations. First, patients and health care providers are not blinded. However, due to the nature of the intervention, blinding is not feasible in this setting. Second, hemodynamic management is not standardized, but remains at the discretion of the treating physician. This is likely to reflect clinical practice and avoids introducing a potential confounder (such as a treatment protocol). Third, the single-center design may limit external validity. However, our unit is a tertiary ICU in a large university hospital and, therefore, is likely to reflect current best practice in the management of critically ill patients with shock.

## Trial status

Patient recruitment has started in May 2014, recruitment will likely be completed by December 2017. Protocol V2 01.12.2013.

## Additional files

Additional file 1: Definitions and measurement of outcome parameters and serious adverse events. (DOCX 44 kb)

Additional file 2: Standard Protocol Items: Recommendations for Interventional Trial (SPIRIT) 2013 Checklist: recommended items to address in a clinical trial protocol and related documents*. (DOC 168 kb)

## Abbreviations

ControlPM: Patient group randomized to control with protocolized monitoring
ControlSM: Patient group randomized to control with standard monitoring
FAC: Fractional area change of left ventricle
hTEE: Qualitative hemodynamic TEE assessment
hTEEPM: Patient group randomized to hTEE with protocolized monitoring
hTEESM: Patient group randomized to hTEE with standard monitoring
ICU: Intensive care unit
LOS: Length of stay
LV: Left ventricle
LVEDA: Left ventricular area at end-diastole
LVESA: Left ventricular area at end-systole
ME asc aortic SAX): Mid-esophageal ascending aortic short-axis view
ME four chamber: Mid-esophageal four chamber view
PDMS: Patient data monitoring system
RV: Right ventricle
RVEDA/LVEDA ratio: Ratio of right ventricular to left ventricular area
SVC CI: Collapsibility index of superior vena cava
SVC: Superior vena cava
TEE: Transesophageal echocardiography
TG mid SAX: Transgastric mid-esophageal short-axis view
