# Correction to: Effects of hemodynamic monitoring using a single-use transesophageal echocardiography probe in critically ill patients – study protocol for a randomized controlled trial

**DOI:** 10.1186/s13063-018-3001-0

**Published:** 2018-10-29

**Authors:** Luca Cioccari, Bjoern Zante, Andreas Bloch, David Berger, Andreas Limacher, Stephan M. Jakob, Jukka Takala, Tobias M. Merz

**Affiliations:** 1 Department of Intensive Care Medicine, University Hospital, and University of Bern, 3010 Bern, Switzerland; 2 CTU Bern, and Institute of Social and Preventive Medicine (ISPM), University of Bern, Bern, Switzerland

## Correction

Following publication of the original article [1], the authors reported an error in the sample size calculation. This correction presents the incorrect, as well as the correct sample size declaration.

Incorrect text:

- “Sample size calculation is based on a retrospective analysis of a sample of 159 patients admitted to our ICU during a 3-month period, which fulfilled the study entry criteria. Median time to resolution of circulatory shock, defined as mean systemic blood pressure > 60 mmHg and resolution of clinical signs of shock (capillary refilling time < 3 s, urine output > 0.5 mL/kg/h for at least 4 h, blood lactate < 2 mmol/L), in this sample was 18.5 h (Fig. 1).”

Correct text:

- “Sample size calculation is based on a retrospective analysis of a sample of 159 patients admitted to our ICU during a 3-month period, which fulfilled the study entry criteria. Median time to resolution of hemodynamic instability as defined by discontinuation of vasopressors or inotropes in this sample was 18.5 hours (Fig. 1).”

The corrected text is identical to the previously published text in the statistical analysis plan on clinicaltrials.gov (https://clinicaltrials.gov/ProvidedDocs/66/NCT02048566/SAP_000.pdf<https://urldefense.proofpoint.com/v2/url?u=https-3A__clinicaltrials.gov_ProvidedDocs_66_NCT02048566_SAP-5F000.pdf&d=DwMFAg&c=vh6FgFnduejNhPPD0fl_yRaSfZy8CWbWnIf4XJhSqx8&r=_Sjd0yL0bevGob014KdftSO3mW49mj-Kr7McyIsCy_Y&m=AeaiTL7BjsfUmXrb_dSMaLQ5Kx9w-0qwysAG5x2SjG0&s=FLSBCcMk0RdwkyPG5QQdZEewtqwDLO1Zt8IuMreqQ4o&e).
