# Supplementary material for: Effects of hemodynamic monitoring using a single-use transesophageal echocardiography probe in critically ill patients – study protocol for a randomized controlled trial
Source: Trials. 2018 Jul 6;19:362. doi: 10.1186/s13063-018-2714-4 (PMC6035404; doi:10.1186/s13063-018-2714-4)
Supplement: Supplementary file 1 — Definitions and measurement of outcome parameters and serious adverse events. (DOCX 44 kb) [file 13063_2018_2714_MOESM1_ESM.docx]

# Additional file 1

**Definitions and measurement of outcome parameters and serious adverse events**

1. Time to resolution of hemodynamic instability

Systemic mean arterial blood pressure (MAP) > 60mmHg for at least 4 hours after discontinuation of vasopressors or inotropes. Data are extracted from the electronic Patient Data Management System (PDMS Centricity Critical Care 7.0 SP2, GE Healthcare, 540 W. Northwest Highway Barrington, IL 60010 USA). To account for artefacts as a source of error, the criterion is considered fulfilled if MAP > 60mmHg for > 90% of the 4 hours requested.

1. Capillary refill time[1]

Good light conditions are ensured before testing. Skin and oral temperature are recorded at the time of measurement. Moderate pressure is applied for at least 3 seconds on the nail of the thumb and big toe, respectively. All four extremities are tested, the best value is recorded by means of a stopwatch (rounded up to seconds). In case of difficulties when performing the measurements, the staff specialist in charge of the patient will repeat the test. The effective value is then determined by agreement.

1. Urine output

Urine output is documented every 2 hours. A mean urine output of > 0.5ml/kg/h during a 4-hour assessment interval is considered as resolution of oliguria.

1. Blood lactate level < 2mmol/l

Blood samples for arterial blood gas analysis and lactate measurement are drawn for study purposes every 2 hours for the first 72 hours after study inclusion or until reversal of circulatory shock as per definition of the primary outcome.

1. Ventricular tachycardia (VT)[2]

In our study, VT is defined as 4 or more consecutive complexes in duration emanating from the ventricles at a rate of greater than 100 bpm (cycle length less than 600 ms), as < 4 ventricular complexes have not been related to an increased risk of sudden cardiac death. Non-sustained VT is VT lasting less than 30 seconds. Sustained VT is present if VT lasts for 30 seconds or more.

1. Ventricular fibrillation[3]

Rapid grossly irregular ventricular rhythm with marked variability in QRS cycle length, morphology, and amplitude.

1. Atrial fibrillation[4]

Replacement of consistent P waves by rapid oscillations or fibrillatory waves that vary in amplitude, shape, and timing, associated with an irregular, frequently rapid ventricular response when atrioventricular conduction is intact.

1. Atrial flutter[4]

Saw-tooth pattern of regular atrial activation called flutter waves on the ECG.[4]

1. Myocardial infarction (MI)[5]

- Elevated cardiac high sensitivity Troponin (hsTnT) values: at least one value above 0.014ug/l (99th percentile) using Electro-Chemiluminescence-Immuno-Assay (Elecsys®, Roche)

AND one of the following:

- symptoms of ischemia
- ischemic ECG changes (pathologic Q, new or presumed new significant ST-segment or T wave changes or new left bundle branch block)
- Identification of an intracoronary thrombus by angiography or autopsy
- Imaging evidence of new loss of viable myocardium or a new regional wall motion abnormality.
- Cardiac death with symptoms suggestive of myocardial ischemia and presumed new ischemia ECG changes or new LBBB.
  1. Percutaneous coronary intervention-related MI[5]
- Elevation of hsTnT > 0.07ug/l (5 x 99th percentile)

OR

- a rise of >20 % if the baseline values are elevated and are stable or falling

AND

- one of the following:
- symptoms of ischemia
- ischemic ECG changes (pathologic Q, new or presumed new significant ST-segment-T wave changes or new left bundle branch block)
- Identification of an intracoronary thrombus by angiography or autopsy
- persistent slow- or no-flow or embolization
- Imaging evidence of new loss of viable myocardium or a new regional wall motion abnormality.
  1. Coronary artery bypass graft surgery-associated MI[5]
- Elevation of hsTnT values > 0.14ug/l (10 x 99th percentile) with normal baseline hsTnT values

AND

- one of the following:
- symptoms of ischemia
- ischemic ECG changes (pathologic Q or new left bundle branch block)
- Identification of an intracoronary thrombus by angiography or autopsy
- Imaging evidence of new loss of viable myocardium or a new regional wall motion abnormality
- angiographic documented new graft of native coronary artery occlusion

1. Acute limb ischemia[6]

Acute onset of at least one of the following unilateral findings:

- Clinical signs: Pallor, cool skin, decreased sensation, decreased strength, absent pulses

AND

- Positive imaging (duplex ultrasonography, computed tomographic angiography, magnetic resonance angiography) or need for urgent revascularization

1. Acute bowel ischemia[7]

- Abdominal tenderness or guarding persisting for more than 2 hours, clinical picture not suggesting other abdominal problem (e.g., cholecystitis or diverticulitis)

AND

- positive findings on CT scan, abdominal angiogram or laparotomy

1. Upper gastrointestinal (GI) bleeding[8]

- Melena
- nasogastric lavage with blood or coffee grounds
- endoscopic findings of upper GI bleeding

1. Lower GI bleeding[9]

Haematochezia associated with

- instability of vital signs
- anaemia
- need for blood transfusion

or endoscopic or angiographic findings of lower GI bleeding

1. Ventilator associated pneumonia[10]

- A Clinical Pulmonary Infection Score (CPIS) > 6 points

1. Catheter related bloodstream infection (CRBSI)[11]

One of the following criteria:

- Culture of the same organism from both the catheter tip and at least one percutaneous blood culture OR
- Culture of the same organism from at least two blood samples (one from a catheter hub and the other from a peripheral vein or second lumen) meeting criteria for quantitative blood cultures or differential time to positivity.
- Quantitative blood cultures demonstrating a colony count from the catheter hub sample ≥3 fold higher than the colony count from the peripheral vein sample (or a second lumen) supports a diagnosis of CRBSI.
- Semiquantitative cultures demonstrating >15 colony-forming units/mL of the same microbe from the insertion site, hub site and peripheral blood culture also supports a diagnosis of CRBSI.
- Differential time to positivity: growth detected from the catheter hub sample at least two hours before growth detected from the peripheral vein sample.

1. Sepsis[12]

Infection, documented or suspected and > 2 SIRS criteria

1. Stroke[13, 14]

- National Institutes of Health Stroke Scale > 1 points AND
- Evidence of ischemic or haemorrhagic stroke by CT, MRI or angiographic imaging

**References**

1. Pickard A, Karlen W, Ansermino JM: **Capillary refill time: is it still a useful clinical sign?** *Anesth Analg* 2011, **113:**120-123.

2. Scirica BM, Braunwald E, Belardinelli L, Hedgepeth CM, Spinar J, Wang W, Qin J, Karwatowska-Prokopczuk E, Verheugt FW, Morrow DA: **Relationship between nonsustained ventricular tachycardia after non-ST-elevation acute coronary syndrome and sudden cardiac death: observations from the metabolic efficiency with ranolazine for less ischemia in non-ST-elevation acute coronary syndrome-thrombolysis in myocardial infarction 36 (MERLIN-TIMI 36) randomized controlled trial.** *Circulation* 2010, **122:**455-462.

3. European Heart Rhythm A, Heart Rhythm S, Zipes DP, Camm AJ, Borggrefe M, Buxton AE, Chaitman B, Fromer M, Gregoratos G, Klein G, et al: **ACC/AHA/ESC 2006 guidelines for management of patients with ventricular arrhythmias and the prevention of sudden cardiac death: a report of the American College of Cardiology/American Heart Association Task Force and the European Society of Cardiology Committee for Practice Guidelines (Writing Committee to Develop Guidelines for Management of Patients With Ventricular Arrhythmias and the Prevention of Sudden Cardiac Death).** *J Am Coll Cardiol* 2006, **48:**e247-346.

4. Fuster V, Ryden LE, Cannom DS, Crijns HJ, Curtis AB, Ellenbogen KA, Halperin JL, Le Heuzey JY, Kay GN, Lowe JE, et al: **ACC/AHA/ESC 2006 Guidelines for the Management of Patients with Atrial Fibrillation: a report of the American College of Cardiology/American Heart Association Task Force on Practice Guidelines and the European Society of Cardiology Committee for Practice Guidelines (Writing Committee to Revise the 2001 Guidelines for the Management of Patients With Atrial Fibrillation): developed in collaboration with the European Heart Rhythm Association and the Heart Rhythm Society.** *Circulation* 2006, **114:**e257-354.

5. Thygesen K, Alpert JS, Jaffe AS, Simoons ML, Chaitman BR, White HD, Katus HA, Lindahl B, Morrow DA, Clemmensen PM, et al: **Third universal definition of myocardial infarction.** *Circulation* 2012, **126:**2020-2035.

6. Creager MA, Kaufman JA, Conte MS: **Clinical practice. Acute limb ischemia.** *N Engl J Med* 2012, **366:**2198-2206.

7. **American Gastroenterological Association Medical Position Statement: guidelines on intestinal ischemia.** *Gastroenterology* 2000, **118:**951-953.

8. Srygley FD, Gerardo CJ, Tran T, Fisher DA: **Does this patient have a severe upper gastrointestinal bleed?** *JAMA* 2012, **307:**1072-1079.

9. Zuccaro G, Jr.: **Management of the adult patient with acute lower gastrointestinal bleeding. American College of Gastroenterology. Practice Parameters Committee.** *Am J Gastroenterol* 1998, **93:**1202-1208.

10. Grgurich PE, Hudcova J, Lei Y, Sarwar A, Craven DE: **Diagnosis of ventilator-associated pneumonia: controversies and working toward a gold standard.** *Curr Opin Infect Dis* 2013, **26:**140-150.

11. Safdar N, Fine JP, Maki DG: **Meta-analysis: methods for diagnosing intravascular device-related bloodstream infection.** *Ann Intern Med* 2005, **142:**451-466.

12. Dellinger RP, Levy MM, Rhodes A, Annane D, Gerlach H, Opal SM, Sevransky JE, Sprung CL, Douglas IS, Jaeschke R, et al: **Surviving sepsis campaign: international guidelines for management of severe sepsis and septic shock: 2012.** *Crit Care Med* 2013, **41:**580-637.

13. Kothari R, Hall K, Brott T, Broderick J: **Early stroke recognition: developing an out-of-hospital NIH Stroke Scale.** *Acad Emerg Med* 1997, **4:**986-990.

14. Moustafa RR, Baron JC: **Clinical review: Imaging in ischaemic stroke--implications for acute management.** *Crit Care* 2007, **11:**227.
